# Radiotherapy-induced vitiligo in a patient with breast cancer, a case report

**DOI:** 10.3332/ecancer.2024.1716

**Published:** 2024-06-20

**Authors:** Francisco E Villanueva, Natalia S Jara, Valentina Darlic

**Affiliations:** 1 Departamento de Dermatología, Facultad de Medicina, Universidad de Chile, Santiago 8380453, Chile; 2 Servicio de Radioterapia, Instituto Nacional Del Cáncer, Santiago 8380455, Chile; 3 Facultad de Medicina, Universidad del Desarrollo, Santiago 7610315, Chile; a https://orcid.org/0000-0003-1003-4565

**Keywords:** vitiligo, radiotherapy, breast cancer, Koebner phenomenon, melanocyte

## Abstract

Vitiligo is a disease characterised by the autoimmune destruction of melanocytes, manifesting as depigmentation of the skin. We present the case of a female patient with a history of breast cancer who developed vitiligo in the area of the treatment field 12 months after the end of radiotherapy. It has been reported in the literature that vitiligo can occur in patients with a history of vitiligo after radiotherapy, attributable to the Koebner phenomenon, where some treatments can induce new vitiligo lesions in the patient.

## Introduction

Vitiligo is a disease that consists of the autoimmune destruction of melanocytes, manifesting as depigmentation of the skin [1, 2]. Its prevalence worldwide is estimated to be between 0.1% and 2%, generating a negative psychological impact on patients [3, 4]. Although genetics plays an important role in the aetiology of vitiligo, immunological, biochemical and environmental factors also affect genetically predisposed patients, making it a multifactorial disease [5, 6].

The following is a clinical case of a patient with a history of vitiligo and breast cancer who, following radiotherapy, presented with depigmentation in the irradiated areas.

## Clinical case

**Figure 1.**
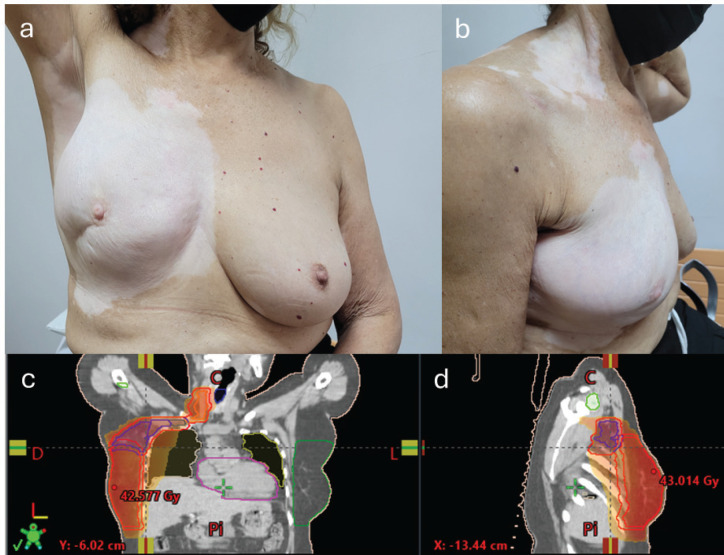
Depigmented spots in the right breast (frontal) (a), right breast and supraclavicular region (lateral) (b) and frontal (c) and lateral (d) radiotherapy treatment field.

A female patient aged 61 years, with a history of arterial hypertension and vitiligo, diagnosed at the age of 40, with a family history of vitiligo (mother). She was diagnosed with triple negative invasive breast carcinoma, stage cT2N1M0. She received four cycles of neoadjuvant chemotherapy (AC scheme doxorubicin and cyclophosphamide). Partial mastectomy plus axillary dissection levels I and II were performed. Subsequently, she received adjuvant external beam radiotherapy on the right breast, plus regional lymph nodes, axillary levels, supraclavicular and ipsilateral internal mammary chain, up to a dose of 50 Gy in 25 sessions. Twelve months after the end of radiotherapy, she reported depigmentation in the irradiated areas. Physical examination revealed depigmented spots in the right breast, axilla, neck, supraclavicular region and dorsum, consistent with the areas where she received radiotherapy (Figure 1).

## Discussion

Vitiligo is an acquired disease, manifested by the appearance of depigmented, asymptomatic, well-defined macules and patches on the surrounding healthy skin. The lesions can appear in any age group and body site, being more frequent around body orifices, genitalia and sun-exposed areas such as the face and hands [7].

Physical, mechanical, chemical/thermal stimuli, allergic or irritant reactions, chronic pressure, inflammatory or therapeutic dermatoses, including radiotherapy, can trigger the development of vitiligo. This is known as the Koebner phenomenon or also called ‘isomorphic response’, which corresponds to the development of lesions on traumatised sites of healthy skin in patients with skin diseases [8]. We suggest that this is the cause of the depigmentation seen after radiotherapy in patients with vitiligo.

This phenomenon has been described in 20%–60% of patients with vitiligo [8, 9].

The time between skin trauma and the Koebner phenomenon has not been studied in patients with vitiligo [8].

There are several theories regarding the pathophysiology of the Koebner phenomenon in vitíligo; however, it is not yet well established. One of these theories is that this phenomenon occurs by immune-mediated mechanisms, where the release of different inflammatory cytokines after skin trauma may activate melanocyte-specific T-cells in the skin and positively regulate their expression of major histocompatibility complex (MHC) and Intercellular Adhesion Molecule 1 (ICAM-1). On the other hand, it has also been described that skin trauma causes increased oxidative stress which directly affects melanocyte function and the loss of melanocytes through phagocytosis by an autoimmune response. Another theory is that in vitiligo there is defective adhesion of melanocytes to fibronectin, which may predispose to pigment cell loss. Finally, it is thought that cutaneous stimuli may decrease the production of growth factors, including stem cell factor, released by keratinocytes, which is necessary for melanocyte survival [8].

Cases of vitiligo have been reported in the literature in patients with a history of vitiligo following radiotherapy. This has been considered in the context of the Koebner phenomenon [10].

A literature review by Mansour *et al* [11] identified 15 studies, describing a total of 18 patients with vitiligo following exposure to radiotherapy. Only 50% of the patients reported a personal history of vitiligo, while 0 patients reported a family history of vitiligo. However, it is noteworthy that only 9 studies refer to family history. Of the total number of patients 13 (72%) were treated with radiotherapy for breast cancer. On average, patients developed vitiligo 5.1 months after completion of radiotherapy. In 89% of cases, depigmentation occurred on the breast/trunk, 28% on the neck, 22% on the face and 22% on the upper extremities. External beam radiotherapy was the only modality used, at a mean radiation dose of 52.3 Gy (range: 40–70) [11].

It has also been suggested that there may be a dose-dependent relationship between radiotherapy and vitiligo. One study reported larger areas of depigmentation in areas of the body that received higher doses of radiation [11, 12].

Therefore, patients with a history of vitiligo should be warned that the Koebner phenomenon may occur after exposure to radiotherapy; however, cases of primary vitiligo have also been reported [10, 11, 13].

However, few cases have been reported with heterogeneous patient characteristics [11].

## Conclusion

Vitiligo can occur in patients with a history of vitiligo after radiotherapy, mainly explained by the Koebner phenomenon. To date, few cases have been reported and further studies are needed to determine the relationship between vitiligo and radiotherapy.

## Conflicts of interest

The authors declare that they have no conflict of interest.

## Funding

The authors have received no financial support for the research, authorship and/or publication of this article.
